# Elevated Plasma Levels of the Pituitary Hormone Cthrc1 in Individuals with Red Hair but Not in Patients with Solid Tumors

**DOI:** 10.1371/journal.pone.0100449

**Published:** 2014-06-19

**Authors:** Christine W. Duarte, J. Patrizia Stohn, Qiaozeng Wang, Ivette F. Emery, Andrew Prueser, Volkhard Lindner

**Affiliations:** Center for Molecular Medicine, Maine Medical Center Research Institute, Scarborough, Maine, United States of America; University of Leuven, Rega Institute, Belgium

## Abstract

**Background:**

An increasing number of studies report that Cthrc1 is expressed in various cancer cells. The present study sought to identify which cells in tumors and remodeling tissues express Cthrc1 and investigate the range of circulating human Cthrc1 levels in health and disease.

**Methodology/Principle Findings:**

Highly specific monoclonal antibodies were generated to detect Cthrc1 by ELISA in plasma and in tissues by immunohistochemistry. In human colon, gastric, breast, endometrial, pancreatic, kidney, lung and skin cancer, Cthrc1 was expressed by activated stromal cells and not the cancer cells themselves. Similarly, conditions evoking tissue remodeling, such as wound repair or angiotensin II-mediated hypertension, induced Cthrc1 expression in interstitial and adventitial fibroblasts and perivascular stromal cells. Levels of Cthrc1 in plasma from healthy subjects were near the lower detection limit except for individuals with red hair, who had up to several hundred fold higher levels. Elevated Cthrc1 was also found in patients with diabetes, inflammatory conditions, and infections, but not solid tumors. Transgenic mouse studies suggested that Cthrc1 expression by stromal cells does not contribute to circulating levels. In human pituitaries, Cthrc1 was expressed in the anterior and intermediate lobes with unencapsulated Cthrc1 accumulations typically surrounded by chromophobe cells.

**Conclusions:**

We identify Cthrc1 as a marker for activated stromal cells. Cthrc1 is a pituitary hormone with significantly elevated levels in subjects carrying variant alleles of the melanocortin-1 receptor as wells as in patients with inflammatory conditions.

## Introduction

We discovered collagen triple helix repeat containing 1 (Cthrc1) in a screen for novel sequences induced in arteries upon balloon catheter injury, where it was prominently induced in activated adventitial fibroblasts [1]. The response to this injury induces smooth muscle cell proliferation with intimal thickening and constrictive remodeling with reduction in lumen size and fibrosis of the adventitia. Since our discovery, Cthrc1 has frequently been identified as a gene upregulated in tumors based on microarray-based screening approaches [2]. Among the cancer cells reported to express Cthrc1 are melanomas [2], [3], hepatocellular carcinoma [4], oral cancer [5], breast ductal carcinoma [6], [7], pancreatic cancer [8], colorectal cancer [9], [10], gastric cancer [11], and dermatofibrosarcoma protuberans [12]. A recent study by Spector et al. [13] reported expression of Cthrc1 in myofibroblasts in muscular dystrophies. Many of the studies reporting Cthrc1 overexpression in cancers relied on microarray expression data and insufficiently characterized antibodies. We previously generated Cthrc1-specific monoclonal antibodies suitable for immunohistochemistry and validated them extensively using tissues from Cthrc1 transgenic mice and Cthrc1 null mice [14]. In the adult, we found expression of Cthrc1 was restricted to the brain, pituitary gland and some osteocytes [14] but expression in parenchymal cells was not detectable. The development of hepatic steatosis in Cthrc1 null mice in the absence of any detectable Cthrc1 expression in the liver led to our discovery that Cthrc1 is a hormone with pituitary and remodeling bone as likely sources contributing to circulating levels.

The present study built on our previous findings of circulating Cthrc1 and sought to investigate the levels of circulating Cthrc1 in healthy human subjects. In addition, approximately 1300 plasma samples from random patients were evaluated for Cthrc1 levels to determine whether Cthrc1 is a biomarker for certain diseases. Furthermore, we used immunohistochemistry performed with rabbit monoclonal antibodies to localize Cthrc1 in various human cancers previously reported to overexpress Cthrc1. To address the question whether overexpression of Cthrc1 in stromal cells leads to elevated circulating levels of Cthrc1, we used a genetic approach with transgenic overexpression of Cthrc1 under the control of the *Pdgfrb* promoter.

## Materials and Methods

### Ethics Statement

All protocols involving animals were approved by the Institutional Animal Care and Use Committee of the Maine Medical Center (protocol number 1204) and were in compliance with all applicable regulations and guidelines including the National Institutes of Health *Guide for Care and Use of Laboratory Animals*. Human plasma samples were obtained under a protocol approved by the Institutional Review Board (IRB) of Maine Medical Center (protocol number 3657). Written consent was obtained from human subjects participating in this study. The IRB had approved the *Informed Consent Form* (ICF) and the procedure used to consent participants for the blood draw. After consenting, the participants were provided with a signed copy of the ICF. The original signed copy of the ICF is kept by the investigators in a secure place.

### Transgenic mice and mouse tissues

Cre-inducible transgenic mice expressing human or rat Cthrc1, Tg(CagGfp-hCthrc1)*^Vli^* and Tg(CagGfp-rCthrc1)*^Vli^,* were generated in the same manner as previously described for the myc-tagged rat Cthrc1 expressing strain Tg(CagGfp-Cthrc1myc)*^11Vli^* [15]. The coding region of human or rat Cthrc1 cDNA (Image clone 4586039, GenBank accession number BC014245) was cloned into the CagGfp vector as described [15] without addition of a tag. These mice were bred with previously described Tg(Pdgfrb-Cre)*^35Vli^* mice [15] expressing Cre recombinase under the control of the *Pdgfrb* promoter. This approach allowed for Cre inducible expression of human Cthrc1 and detection of circulating Cthrc1 with the human specific enzyme linked immunosorbent assay (ELISA) described below.

Paraffin-embedded, formalin-fixed tissue blocks were prepared from pancreatic ductal adenocarcinoma (kindly provided by Dr. Howard Crawford, Mayo Clinic, Jacksonville, FL), testicular germ cell tumor, skin wounds, ischemic muscle tissue, anti-collagen antibody-induced arthritic joints [16], and hearts and arteries from wildtype and Cthrc1 null mice mice infused with angiotensin II for 7 days (500 ng/kg/min via osmotic minipump) [14].

### Human plasma samples and paraffin-embedded tissue blocks

Samples were obtained from healthy adult male and female volunteers after obtaining informed consent. In collaboration with a primary care facility 792 plasma samples with EDTA as anticoagulant were obtained from patients visiting their primary care physicians. In addition, Maine Medical Center's BioBank, a core facility of the institution, provided us with 505 plasma samples with lithium heparin as anticoagulant from hospitalized patients. All plasma samples would have otherwise been discarded. Plasma was processed and frozen within 6 hours of the blood draw for the outpatient samples and within 24 hours for the inpatient samples. After plasma isolation all samples were stored at −70°C until analysis.

Maine Medical Center's BioBank also provided us with de-identified formalin-fixed, paraffin-embedded tissue blocks of various types of cancers that were reported to frequently overexpress Cthrc1 [2]. These included melanomas (n = 10), breast cancer (n = 10), colon cancer (n = 10), gastric cancer (n = 10), pancreatic cancer (n = 10), lung cancer (n = 5) and uterine cancer (n = 5). Human pituitary glands were obtained as autopsy specimens and the average ischemia time until fixation for those was 25 hours.

### Monoclonal antibody and ELISA development

**Figure 1:**
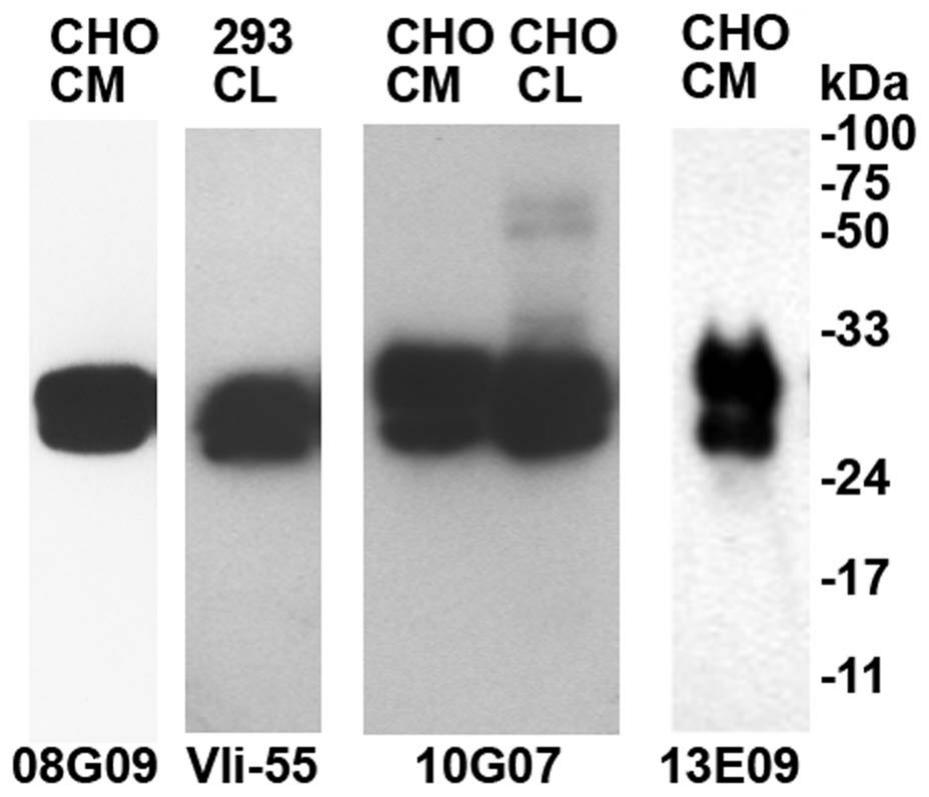
Characterization of anti-Cthrc1 monoclonal antibodies. HEK293T cells (293) and CHO-K1 cells (CHO) were transduced with human or rat Cthrc1 expressing adenovirus. Cell lysates (CL) or conditioned media (CM) were collected for immunoblot using four different antibodies as indicated. The double band recognized by all the antibodies represents Cthrc1 with partially cleaved signal peptide as determined by mass-spectrometry (data not shown). Mouse monoclonal antibody 08G09 reacts with rat and mouse Cthrc1 but not human, rabbit monoclonal Vli-55 is specific for the conserved C terminus present in all species, mouse monoclonal 10G07 detects only the N terminal epitope of human Cthrc1, mouse monoclonal antibody 13E09 detects an internal epitope present in mouse, rat and human Cthrc1.

BALB/c and Cthrc1 null mice on the C57BL/6 [14] background were immunized with synthetic peptides corresponding to the N terminus of human and murine Cthrc1 (SENPKVKQKAQLRQRE and SENPKVKQKALIRQRE) coupled to keyhole limpet hemocyanin as well as recombinant rat Cthrc1 with C terminal 8xHis expressed in *E.coli*. Using the services of Maine Biotechnology Services (Portland, ME), hybridomas were generated. Hybridoma supernatants were screened by indirect ELISA against human and rat Cthrc1 expressed in CHO-K1 cells. In addition, the supernatants were also screened by indirect ELISA against the N terminal peptides conjugated to bovine serum albumin (BSA) as well as by immunoblot analysis of rat and human Cthrc1. All monoclonal antibodies referenced here are made available through www.mmcri.org/antibody. They detected a double band of approximately 30 kDa and 32 kDa on immunoblots of lysates and conditioned media of Cthrc1 adenovirus transduced CHO-K1 cells run under reducing conditions (Figure 1). Mass-spectrometry identified the upper band as Cthrc1 with the signal peptide still present and the lower band as Cthrc1 with the signal peptide cleaved (data not shown). Monoclonal antibody 10G07 is specific for the N terminus of human Cthrc1 and does not react with rat or murine Cthrc1. Clones 02F06, 08G09, 16B04 are specific for the N terminus of rat/mouse Cthrc1 and do not react with human Cthrc1 (not shown). Clone 13E09 recognizes an epitope located within the N terminal half of the molecule of both human and rodent Cthrc1 (not shown).

Clone 13E09 is suitable as a capturing antibody in a sandwich ELISA and biotinylated 10G07 or 08G09 (OneQuant Sulfo-NHS-LC Biotin, G-Biosciences) were used as detection antibodies for human and rodent Cthrc1, respectively. It is important to note that none of the monoclonal antibodies generated recognized C terminally modified Cthrc1 by ELISA, even though their epitopes mapped to the N terminal half of the molecule. This included Cthrc1 with C terminal myc tag, myc/6xHis tag, 8xHis tag, or Cthrc1 with deletion of the C terminal lysine (K) residue.

Cthrc1 standards were prepared from purified Cthrc1 with a 7xHis tag fused to the N terminus and expressed in CHO-K1 cells. For analysis of plasma samples for Cthrc1 levels, ELISA plates (MAXISORP, Nunc) were coated at 4°C overnight with 100 µl of anti-Cthrc1 13E09 at 1.8 µg/ml in carbonate buffer (pH = 9.6). Plates were washed four times between all incubations with 400 µl per well of PBS-T containing 0.1% BSA. Wells were blocked with 400 µl of PBS-T containing 0.1% BSA for 1 hour at room temperature on a plate shaker. Samples of 100 µl of undiluted plasma were incubated for 2 hours at room temperature, followed by incubation with biotinylated anti-Cthrc1 10G07 used at 0.5 µg/ml for 60 min. Streptavidin-HRP (Vector Laboratories) was used at 1∶1000 dilution followed by the colorimetric reaction with TMB substrate (TMB PLUS LIQUID, Amresco). Optical densities were read at 450 nm with a plate reader (Apollo LB913, Berthold Technologies). The analytical sensitivity of the assay was determined to be 75 pg/ml by calculating the mean ± 3 standard deviations for 16 replicates of the assay diluent. The lower limit of detection of the assay is 160 pg/ml.

### Immunohistochemistry, immunoblotting and cell culture

Rabbit monoclonal anti-Cthrc1 clone Vli-55 (www.mmcri.org/antibody) was used for Western blotting and immunohistochemistry on paraffin-embedded, formalin-fixed tissues at 20 ng/ml following antigen retrieval with citrate buffer (0.1 M, pH = 6.0, 10 min in a steamer). This antibody had been previously characterized. It shows no immunoreactivity in any tissue of Cthrc1 null mice [14]. Subsequent steps of the immunostaining procedure were executed as previously published [14]. For immunostaining on tissues containing melanin-expressing cells a red color reaction product was chosen using a kit (VECTASTAIN ABC alkaline phosphatase, VECTOR Red Alkaline Phosphatase Substrate, Vector Laboratories). None of the mouse monoclonal antibodies were suitable for immunohistochemistry on paraffin sections. Immunoblotting was performed on cell lysates (CL) and conditioned media (CM) of HEK293T cells (293) and CHO-K1 cells (CHO) transduced with rat or human Cthrc1 expressing adenovirus as described [14]. Cthrc1 monoclonal antibodies were used at 10 ng/ml.

### Statistical Analyses

Data were analyzed using the R programming language. Univariate association tests were performed using t-tests for continuous variables and using chi-squared tests for categorical variables. Cthrc1 levels were set to zero if below the detection limit. Another set of univariate analyses were performed in which a dichotomized version of Cthrc1 (either detectable or >0 or not detectable) was associated with patient characteristics either using a t-test or a chi-squared test.

For association of Cthrc1 levels with clinical diagnoses, because of the non-normal distribution of Cthrc1 levels, a non-parametric Wilcoxon rank sum test was used to measure the association between Cthrc1 levels and particular diagnoses. For Maine Medical Center inpatient data, EPIC electronic medical record system codes were extracted from the patient's history and for each ICD-9 (International Classification of Disease) code, a patient was listed as having or not having that diagnosis. Then a Wilcoxon test was used to find the association between Cthrc1 levels for ICD-9 codes with at least 5 patients. Another set of analyses were run in which ICD-9 codes were grouped at the level before the decimal point. For the outpatient samples from the primary care facility where EPIC codes were not available, the doctor's notes were parsed and searched for common medical conditions taking into account misspellings or common synonyms (diabetes, type II diabetes, T2D, etc.). Again, for each condition, patients were divided into categories with and without the condition, and a Wilcoxon test was used to test association with Cthrc1 levels.

## Results

### Cthrc1 expression in remodeling tissues of adult mice

**Figure 2:**
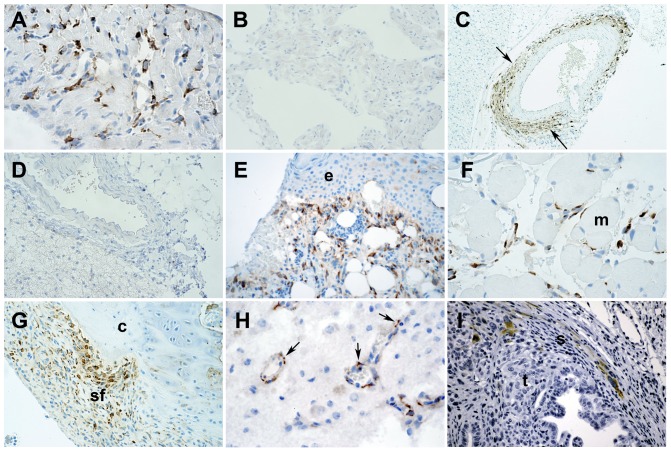
Immunolocalization of Cthrc1 in various remodeling mouse tissues. Immunohistochemistry was performed using rabbit monoclonal antibody Vli-55. (A) In response to angiotensin II, Cthrc1 is induced in interstitial myocardial cells of wildtype mice, but no expression is detected in similarly treated Cthrc1 null mice (B). (C) Angiotensin II-induced Cthrc1 in outer mural cells of the renal artery of wildtype mice (arrows) but not Cthrc1 null mice (D). (E) A skin incisional wound with Cthrc1 localization in activated dermal cells but not in the epidermis (e). (F) Ischemic muscle injury led to Cthrc1 protein expression in interstitial cells but not myocytes (m). (G) An arthritic joint with Cthrc1 in activated synovial fibroblasts (sf) but not in chondrocytes (c) is shown. (H) Remodeling tumor vessels of a germ cell tumor reveal Cthrc1 in outer mural cells (arrows), and (I) pancreatic ductal cancer shows Cthrc1 in cells of the surrounding stroma (s) but not in the tumor cells (t). Cthrc1 immunoreactivity in brown with hematoxylin counterstain. Original magnification 100x (C), 200x (B, D, E, G, I), and 400x (A, F, H).

Increased Cthrc1 expression has been reported in many cancer cells using a variety of commercially available polyclonal antibodies, however, most of them lack rigorous characterization with respect to specificity in immunohistochemistry applications. We used tissues from wildtype and Cthrc1 null mice to evaluate specificity of Cthrc1 immunohistochemistry. Among the many polyclonal and monoclonal anti-Cthrc1 antibodies generated in our laboratory, only clone Vli-55 was found to be specific for immunohistochemistry applications (Fig. 2). Wildtype mice but not Cthrc1 null mice had Cthrc1 protein localization in interstitial cells and myofibroblasts of remodeling tissues, such as the heart and arteries in response to continuous angiotensin II infusion (Fig. 2A-D). These findings are consistent with our original discovery of Cthrc1 by in situ hybridization in the remodeling adventitia of balloon injured rat arteries [1]. Similarly, skin incisional wounds (Fig. 2E) and ischemic muscle injury induced by femoral artery ligation (Fig. 2F) showed Cthrc1 protein in interstitial cells and activated fibroblasts but no expression was detectable in uninjured tissues (not shown). In a model of rheumatoid arthritis induced by anti-collagen antibody injection [16], Cthrc1 protein was localized in activated synovial fibroblasts (Fig. 2G). In a spontaneously occurring testicular germ cell tumor, Cthrc1 was detected in outer mural cells of remodeling small vessels (Fig. 2H). In pancreatic ductal carcinoma, Cthrc1 was localized in some stromal cells surrounding the cancer cells (Fig. 2I). In none of the specimens examined did we ever find Cthrc1 in the parenchymal cells or the tumor cells themselves.

### Cthrc1 in stromal cells of human cancers

**Figure 3:**
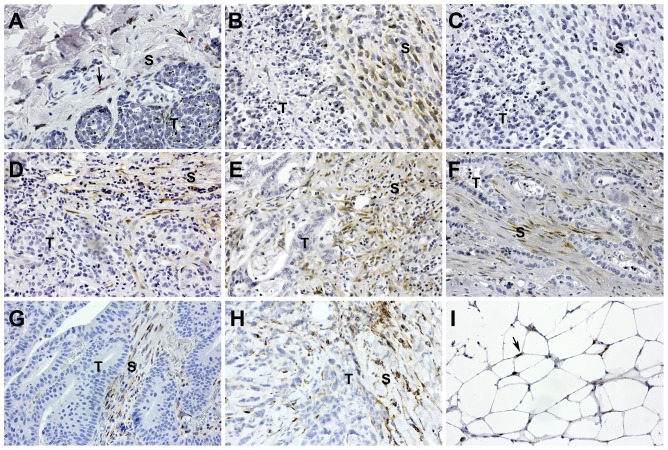
Cthrc1 localizes to tumor stroma. Immunolocalization of Cthrc1 was performed in various human cancers to determine protein levels in stromal cells (S) and tumor cells (T). (A) Melanoma biopsies showed few Cthrc1-positive stromal cells surrounding the tumor (red stain, arrows). (B) Endometrial cancer had abundant Cthrc1 positive stroma adjacent to necrotic tumor area. (C) The endometrial cancer was stained with an irrelevant rabbit monoclonal antibody to ensure specificity. Tumor stroma from (D) breast ductal carcinoma, (E) colon adenocarcinoma, (F) pancreatic adenocarcinoma, and (G, H) lung carcinoma had abundant Cthrc1 protein localization surrounding the tumor cells. (I) Some adipocytes adjacent to the tumor had Cthrc1 expression (arrows). Cthrc1 immunoreactivity in brown (except A) with hematoxylin counterstain. Original magnification 200x.

Microarray analyses have identified Cthrc1 as a gene overexpressed in many cancers [2], [3] [4] [5], [6], [7], [8], [9], [10], [11], [12] with reports of Cthrc1 localization in cancer cells as determined by immunohistochemistry. It does not appear that the specificity of any commercial antibody was verified using negative control tissues from Cthrc1 null species. Our inability to detect Cthrc1 in the parenchyma of tissues prompted us to investigate Cthrc1 expression in various human cancers by immunohistochemistry using rabbit monoclonal anti-Cthrc1 Vli-55. We were unable to detect Cthrc1 expression in any of the cancer cells themselves, but invariably observed Cthrc1 expression in stromal cells within the tumor or surrounding tumor cells (Fig. 3A-H). Occasionally white adipocytes adjacent to the tumor also expressed Cthrc1 (Fig. 3I).

### Circulating levels of Cthrc1

We recently reported that Cthrc1 is a circulating factor detectable qualitatively in plasma of healthy human subjects [14]. Here we established a sensitive sandwich ELISA for quantification of Cthrc1 levels in plasma. Because hitherto nothing is known about Cthrc1 blood levels, we sought to determine Cthrc1 levels in healthy human subjects initially only with respect to gender and age. Furthermore, we wanted to know whether any conditions or diseases are associated with altered Cthrc1 levels.

**Figure 4:**
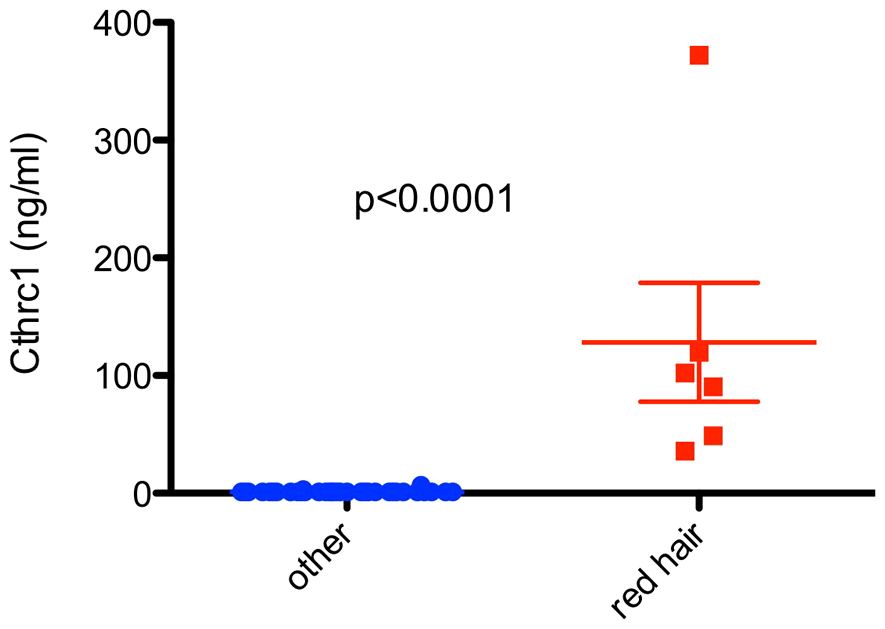
Cthrc1 plasma levels in healthy human subjects. Significantly higher Cthrc1 plasma levels were found in all subjects with red hair.

In most healthy human subjects Cthrc1 levels were close to the lower limit of detection of the assay (160 pg/ml) (Fig. 4) with very few exceptions. It turns out, that all healthy subjects with elevated Cthrc1 levels shared the trait of red hair and fair skin that tans poorly (Fig. 4). It is well established that individuals with red hair and/or poorly tanning skin are carriers of variants of the melanocyte-stimulating hormone receptor gene (MC1R) [17], [18].

Table 1 shows the basic clinical and demographic characteristics of our patient population. Our patient samples came from two clinical sites: Maine Medical Center Inpatient Unit (N = 505), and an outpatient primary care facility in southern Maine (N = 792), as well as a set of healthy volunteer controls (N = 40). The Cthrc1 levels varied across different sites. Higher Cthrc1 levels were seen at the outpatient location (median concentration of 41.9 ng/mL as opposed to 12.5 ng/mL at MMC) potentially affected by the time to processing and type of anticoagulant used (see details in Methods). The normal controls had a high average concentration, but this was accounted for by the disproportionate overrepresentation of red haired individuals (Fig. 4). Cthrc1 plasma levels for healthy individuals excluding this sub-population were below the detection limit of the assay. For this reason, comparisons in Cthrc1 levels need to be made within and not between patient data sets.

**Table 1:** Basic characteristics of the patient population used in this study.

	MMC Inpatients (N = 505)	Primary Care Outpatients (N = 792)	Healthy Controls (N = 40)
**Cthrc1**			
Detectable	163 (32.3%)	646 (81.6%)	8 (20%)
Not Detectable	234 (46.4%)	131 (16.5%)	32 (80%)
Missing	108 (21.4%)	15 (1.9%)	0 (0.0%)
Cthrc1 concentration (ng/mL) in samples with detectable levels median (IQR)	12.5 (25.5)	41.9 (97.0)	69.6 (78.2)
**Gender**			
Male	263 (52.2%)	355 (44.8%)	13 (32.5%)
Female	241 (47.8%)	437 (55.2%)	26 (65.0%)
Missing	1 (0.2%)	0 (0.0%)	1 (0.025%)
**Age** (years)	52 (20)	62 (24)	41 (18)
Missing	1 (0.2%)	0 (0.0%)	3 (7.5%)
**Height** (inches)	66.7 (7)	-	66 (4)
Missing	53 (10.5%)	-	3 (7.5%)
**Weight** (pounds)	188.7 (65.3)	-	142.5 (36.3)
Missing	48 (9.5%)	-	4 (10%)
**Body Mass Index**	29.3 (10.6)	29.5 (8.9)	24.2 (2.6)
Missing	54 (10.7%)	293 (37.0%)	14 (35%)
**Hair color**			
Red	-	-	6 (15%)
Not red	-	-	32 (80%)
Missing	-	-	2 (5%)

Summary statistics by site are shown as median (interquartile range) for continuous variables and number (percentage) for categorical variables. A missing value of Cthrc1 indicates that the ELISA could not be performed due to insufficient sample volume. Statistical tests involving Cthrc1 were performed with samples with non-missing Cthrc1 readings; summary statistics for demographic samples were given for all samples.

Table 2 shows the association between patient characteristics and levels of Cthrc1 (either >0 or detectable or not detectable). We show a strong association between high Cthrc1 levels and red hair color (p = 1×10^−5^). In addition we show an association between increased BMI and high levels of Cthrc1 in the outpatient group (p = 5.93×10^−4^), however, this association was not observed in either the inpatient or healthy controls group. Age and gender did not show an association with Cthrc1 levels.

**Table 2:** Univariate association of demographic variables with Cthrc1.

	MMC Inpatients (N = 505)		Primary Care Outpatients (N = 792)		Healthy Controls (N = 40)	
	*Cthrc1>0*	*N.D.*	*Cthrc1>0*	*N.D.*	*Cthrc1>0*	*N.D.*
**Gender**		*p* = 0.76		*p* = 0.70		*p* = 0.66
Male	80 (49.1%)	119 (50.9%)	288 (44.6%)	61 (46.6%)	3 (42.9%)	10 (31.3%)
Female	83 (50.9%)	115 (49.1%)	358 (55.4%)	70 (53.4%)	4 (57.1%)	22 (68.8%)
**Age**	48.6	50.1 (0.29)	60.0	58.7 (0.44)	38.6	39.4 (0.883)
**Height (inches)**	66.5	66.3 (0.79)	-	-	68.4	66.1 (0.158)
**Weight (pounds)**	190.7	196.4 (0.36)	-	-	165.0	148.3 (0.341)
**Body mass index**	31.0	31.8 (0.59)	**30.9**	**28.6 (5.93e-4)**	26.8	23.7 (0.209)
**Hair Color**		-		**-**		***p*** ** = 1.0e-5**
Red	-	-	-	-	**6 (75%)**	**0 (0%)**
Not red	-	-	-	-	**2 (25%)**	**30 (100%)**

For categorical variables, percentage is given for each group (Cthrc1 > 0 or not detectable, N.D.) and *p*-value is from the Fisher's exact test. For continuous variables, the mean value for each group is given with the p-value from a t-test given in parentheses. Significant associations are shaded in grey.

Tables 3 and 4 show the association between clinical diagnostic categories and high Cthrc1 levels in the MMC inpatient group for minor and major ICD-9 groupings, respectively. These groupings define whether a patient had a given diagnosis, defined by ICD-9 code in EPIC, recorded in their electronic medical record. The total number of different ICD-9 categories within our populations was 134 and 130 for minor and major categories, respectively. Categories shown have a p-value < 0.05 and the estimated false discovery rate (FDR) is given. We identified 12 categories associated with higher levels of Cthrc1 in the minor grouping category (Table 3), and 10 categories associated with higher levels of Cthrc1 in the major grouping category (Table 4). Of note are the presence of blood disorders (acute myeloid leukemia, anemia, volume overload), the presence of infectious or inflammatory conditions (fever, thrush, transaminitis, colitis, urinary tract infection), and particularly diabetes, which is also considered an inflammatory condition [19].

**Table 3:** Univariate association of EPIC diagnostic codes (ICD-9 codes) with Cthrc1 for MMC inpatient data.

ICD-9	Diagnosis	With Diagnosis	Mean without Diagnosis	Mean with Diagnosis	Test Statistic	p-value	FDR
205.00	acute myeloid leukemia	7	14.21	29.06	1799.50	0.0022	0.1478
276.69	volume overload	6	14.54	33.30	1545.00	0.0027	0.1478
780.60	fever	26	12.95	13.48	4673.50	0.0033	0.1478
112.0	thrush	9	14.04	25.17	1912.00	0.0044	0.1478
285.9	anemia	68	12.02	33.96	9115.50	0.0083	0.1968
285.21	anemia in end-stage renal disease or chronic kidney disease	9	14.78	43.49	1823.50	0.0088	0.1968
780.57	sleep apnea	13	12.14	70.76	2325.50	0.0115	0.2211
250.60	diabetic neuropathy or unspecified type diabetes mellitus with neurological manifestations	15	14.13	60.12	2805.00	0.0213	0.3566
599.0	urinary tract infection	19	12.29	70.35	2978.50	0.0270	0.4023
250.01	diabetes mellitus, type 1	6	14.87	18.91	1462.00	0.0352	0.4268
008.45	clostridium difficile colitis	7	10.34	41.99	1297.00	0.0373	0.4268
790.4	transaminitis	10	12.38	78.87	1668.00	0.0382	0.4268

A Wilcoxon rank sum test is used to compare the rank of Cthrc1 levels for patients with and without a given ICD-9 code. Only codes with at least 5 patient instances are included in the analysis. Included results are the mean in each group, the Wilcoxon test statistic, the p-value, and the adjusted p-value (the estimated false discovery rate, FDR). Results presented have a p-value <0.05 and the estimated FDR is shown. ICD-9 codes and a representative diagnosis are shown.

**Table 4:** Univariate association of EPIC diagnostic codes (ICD-9 codes) with Cthrc1 for MMC inpatient data, summarized at the major grouping (before the decimal point).

ICD-9	Diagnosis	With Diagnosis	Mean without Diagnosis	Mean with Diagnosis	Test Statistic	p-value	FDR
998	wound infection after surgery	6	13.54	64.09	1560.50	0.0021	0.2788
112	yeast infection	16	16.20	25.89	2918.50	0.0092	0.5470
v70	healthcare maintenance	39	14.61	24.86	5189.00	0.0226	0.5470
285	anemia	81	12.56	17.76	9878.50	0.0280	0.5470
327	obstructive sleep apnea	20	14.43	33.41	3052.00	0.0296	0.5470
599	urinary tract infection	26	13.16	55.53	3735.00	0.0320	0.5470
8	clostridium difficile colitis	7	10.34	41.99	1297.00	0.0373	0.5470
707	ulcer	19	12.04	53.32	3350.00	0.0416	0.5470
v22	pregnant state	10	14.66	38.09	1823.00	0.0421	0.5470
799	hypoxia	17	15.34	37.08	2330.50	0.0498	0.5530

A Wilcoxon rank sum test is used to compare the rank of Cthrc1 levels for patients with and without a given ICD-9 code. Only codes with at least 5 patient instances are included in the analysis. Included results are the mean in each group, the Wilcoxon test statistic, the p-value, and the adjusted p-value (the estimated false discovery rate). Results presented have a p-value <0.05 and the estimated FDR is shown. ICD-9 major codes and a representative diagnosis are shown.

In comparing the results of the MMC inpatient set to the primary care facility outpatient set, a number of similarities were observed (see Table 5). In this data set, in which 96 conditions were tested, the obesity and inflammation-related disorders are found to be significant (see diagnosis of diabetes, obesity, hyperlipidemia, hyperglycemia in Table 5). In summary, analyses of randomly selected patients show that significantly elevated Cthrc1 levels were found in patients with diabetes, infections or inflammatory and febrile conditions, as well as anemia and leukemia (Tables 3, 4 and 5). In addition, pregnancy was also associated with elevated Cthrc1 levels. However, notably, Cthrc1 levels in patients with solid tumors were not significantly elevated. In particular, for the minor groupings, 162.9 (lung cancer) had six individuals with no significant association with Cthrc1 levels (p = 0.68, data not shown), and a personal history of a number of cancers (including malignant neoplasm of the bronchus and lung v10.11, cervical cancer v10.41, testicular cancer v10.47, ovarian cancer v10.43, laryngeal cancer v10.21, melanoma v10.82, prostate cancer v10.46, and rectal cancer v10.06) in the major groupings with 9 patients showed no significant association (p = 0.95, data not shown). In the outpatient data set, adenocarcinoma, which had 19 patients, was found to be not significant (p = 0.67, data not shown) and pulmonary nodule, which had 12 patients was also found to be not significant (p = 0.69, data not shown).

**Table 5:** Univariate association of common phrases from the patient history coded for patients from the primary care facility data set with Cthrc1.

Diagnosis	With Diagnosis	Mean without Diagnosis	Mean with Diagnosis	Test Statistic	p-value	FDR
diabetes	264	70.47	123.77	79613	0.000029	0.001659
type 2 diabetes	184	75.77	129.85	65115	0.000035	0.001659
hypertension	417	73.46	101.63	86731.5	0.000088	0.002829
osteoporosis	40	87.52	108.04	17576.5	0.019877	0.398531
spinal stenosis	25	85.38	184.77	11645.5	0.020757	0.398531
hyperlipidemia	313	81.53	99.02	78462	0.028095	0.430823
hyperglycemia	23	88.30	97.61	10591.5	0.034756	0.430823
erectile dysfunction	63	86.57	111.34	25542	0.036680	0.430823
hyperthyroidism	6	88.23	132.99	3267.5	0.040390	0.430823
obesity	116	84.13	113.95	42074	0.046534	0.446724

A Wilcoxon rank sum test is used to compare the rank of Cthrc1 levels for patients with and without a phrase found in their history. Only phrases with at least 5 patient instances are included in the analysis. Included results are the mean in each group, the Wilcoxon test statistic, the p-value, and the adjusted p-value (the estimated false discovery rate). Results shown have a p-value <0.05 and the estimated FDR is shown.

### Potential sources of circulating Cthrc1

**Figure 5:**
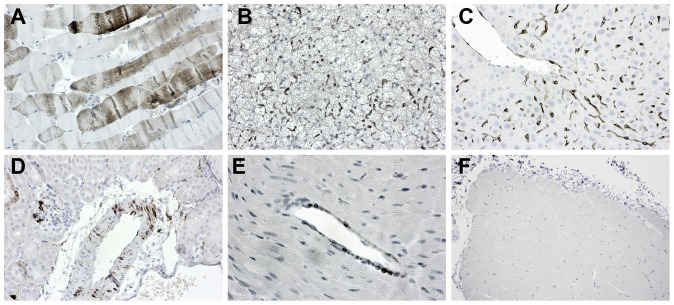
Mouse transgenic model of Cthrc1 overexpression. Cthrc1 protein was assessed by immunohistochemistry in bi-transgenic Tg(CagGfp-hCthrc1)*^Vli^/* Tg(Pdgfrb-Cre)*^35Vli^* mice. (A) Skeletal muscle, (B) capillaries in brown adipose tissue, (C) hepatic endothelium, (D) vascular smooth muscle in the kidney, and (E) mural cells of venules expressed Cthrc1. (F) As a control, no Cthrc1 expression is detectable in wildtype skeletal muscle. Cthrc1 immunoreactivity in brown with hematoxylin counterstain. Original magnification 200x (A-D), 400x (E), and 100x (F).

We previously described transgenic mice overexpressing myc-tagged Cthrc1 under control of the CMV promoter and observed circulating levels of Cthrc1 in plasma that were readily detectable by immunoblotting with HRP-conjugated anti-Cthrc1 antibodies [14]. In these mice, Cthrc1 was predominantly ectopically expressed in the liver, indicating that expression by hepatocytes will result in circulating Cthrc1 levels. With Cthrc1 expression in wound healing and cancers originating from activated stromal cells we wanted to determine whether Cthrc1 expressed by these cells can account for circulating levels with potential systemic effects. Because Pdgfrb is expressed in mesenchymal and stromal cells [20], we used a transgenic mouse expressing Cre recombinase under control of the *Pdgfrb* promoter [15] to overexpress Cre inducible human Cthrc1. This allowed us to determine plasma levels specifically for human Cthrc1 because our ELISA for Cthrc1 does not detect murine Cthrc1. In bi-transgenic Tg(CagGfp-hCthrc1)*^Vli^/* Tg(Pdgfrb-Cre)*^35Vli^* mice Cthrc1 was overexpressed in skeletal muscle (Fig. 5A), small vessels of adipose tissue and liver (Fig. 5B, C) as well as vascular smooth muscle (Fig. 5D). The transgene was also expressed in outer mural cells (Fig. 5E) similar to endogenous Cthrc1 in remodeling vessels (Fig. 2C and H). Analysis of Cthrc1 plasma levels by ELISA, however, revealed that transgenic overexpression did not increase circulating Cthrc1 levels as they remained below the detection limit in both transgenic and wildtype mice (data not shown). This suggests that Cthrc1 expressed in these tissues may not be contributing to circulating levels and instead is likely be acting locally.

**Figure 6:**
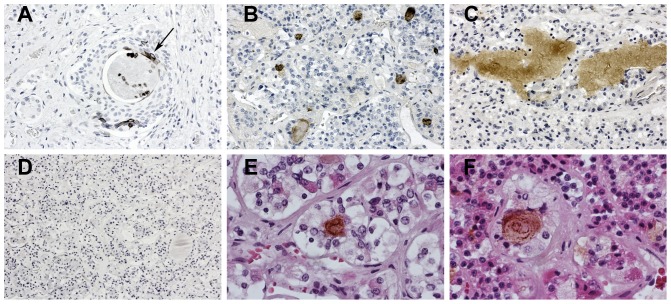
Cthrc1 localizes to the pituitary gland. Immunolocalization of Cthrc1 in human pituitary glands identifies cells expressing Cthrc1 in the anterior lobe (A, arrow). (B, C) Accumulations of colloid-like material contain Cthrc1 immunoreactivity. (D) Absence of non-specific staining is observed with an irrelevant monoclonal antibody. (E, F) Cells surrounding Cthrc1 immunoreactivity are of the chromophobe type. Cthrc1 immunoreactivity in brown with hematoxylin counterstain. Original magnification 200x (A-C), 100x (D), 400x (E, F).

Recently we reported expression of Cthrc1 in the anterior pituitary of rats and pigs [14]. Here we obtained human pituitary glands and localized Cthrc1 in them by immunohistochemistry. Localized accumulations and small lacunae of Cthrc1 immunoreactivity were found throughout the anterior lobe (Fig. 6). In most cases these accumulations were surrounded by chromophobe cells but they were not encapsulated. Cytoplasmic immunoreactivity indicating expression by cells was clearly evident (Fig. 6A). These findings clearly identify Cthrc1 as a pituitary factor in humans.

## Discussion

The present study had several purposes. They included i) identification of cells expressing Cthrc1 in cancers and normal tissues, ii) determination of circulating levels of Cthrc1 and identify conditions associated with altered Cthrc1 levels, iii) characterization of Cthrc1 plasma levels as a potential biomarker, and iv) identification of cell types contributing to circulating levels of Cthrc1. Using rigorously validated monoclonal Cthrc1 antibodies we demonstrate that in all of our cases Cthrc1 expression in human cancers originates from activated stromal cells, not the tumor cells themselves. As we have shown earlier [1] and recently confirmed by Spector et al [13], Cthrc1 is characteristically expressed by the activated fibroblast in remodeling, non-cancerous tissues. In novel Cre-inducible transgenic mice that overexpress Cthrc1 in cells where the *Pdgfrb* promoter had previously been activated, we found no elevation of circulating Cthrc1 levels. This suggests that expression of Cthrc1 by a variety of cell types including vascular cells, skeletal muscle cells and fibroblasts, is not likely to contribute to circulating Cthrc1 levels. Instead, Cthrc1 expressed by these cells may be acting locally in a paracrine or autocrine manner. This idea is supported by the fact that we did not observe elevated Cthrc1 levels in patients with solid cancers. With regards to the cancer patient population one limitation is that our analysis focused on general patients and not specifically oncologic patients, and thus we did not have high power to detect association with many cancers. In the major groupings analysis, the group that contains AML (acute myelogenous leukemia 205.00 and chronic myeloid leukemia 205.10) just barely missed the cutoff (9 cases, p = 0.0523, FDR = 0.55) but was generally consistent with the minor groupings analysis. Future work with a focus on oncology patients is required for a more in-depth investigation of Cthrc1 levels in cancer patients.

A common theme emerged with the detection of elevated Cthrc1 levels in patients suffering from conditions that have an inflammatory or infectious component, e.g. fever, yeast and urinary tract infections, thrush, etc., and even diabetes can be included in this category [19]. In this context it should be mentioned that the promoter region of Cthrc1 contains a putative NF-κB binding site located within 300 base pairs upstream of the transcriptional start site, however, its function has until now not been examined. Combined these findings suggest a link between Cthrc1 levels and inflammation.

Examining Cthrc1 plasma levels in healthy human subjects we made the unexpected discovery that all subjects with red hair had elevated Cthrc1 levels, some with levels several hundred fold higher than subjects with hair color other than red. Valverde et al. [17] have demonstrated previously that variants of the MC1R gene are responsible for pigmentation and red hair color in humans. Signaling via MC1R is responsible for the production of eumelanin (black pigmentation) and three variant alleles account for 60% of all red hair cases in humans [18]. Among Europeans approximately 4% of the population has red hair with the highest percentage of approximately 13% found in Scotland [21] **.** While MC1R variants are under selective pressure in Africa, Harding et al. [22] found no evidence for selection in European populations despite the increased risk of skin cancer [23], [24]. Our study is the first to identify a hormonal factor, Cthrc1, to be elevated in subjects with red hair. We have demonstrated that deletion of the Cthrc1 gene leads to fatty liver (steatosis) formation in mice [14] while others showed that inactivation of this gene also results in low bone mass [25]. The relationship between impaired MC1R signaling and elevated Cthrc1 levels with potential consequences for bone and liver requires further investigation. Ligands for the melanocortin receptors, such as alpha-melanocyte-stimulating hormone (α-MSH) are derived from the POMC precursor protein through processing by prohormone convertases. Originally discovered in the pituitary gland, α-MSH plays an important role in energy balance as well as inhibition of inflammation mediated predominantly via inhibition of NF-κB activation [26], [27], [28]. MC1R is not only expressed on melanocytes but also on non-melanocytic cutaneous cells as well as on cells regulating immune responses and inflammation, e.g. gut mucosa, dendritic cells, macrophages, mast cells and neutrophils (for review [29]). Furthermore, MC1R is also expressed on chondrocytes [30] suggesting a role in bone formation. Currently no information is available for humans carrying variant alleles of MC1R with regards to inflammatory status, and any potential link between melanocortin receptor signaling and Cthrc1 expression requires further investigations.

In the process of generating monoclonal antibodies recognizing unmodified Cthrc1 in its native form by ELISA, we made the important observation that any C terminal modification of Cthrc1 interfered with detection by the antibody. The inability to recognize Cthrc1 modified with C terminal epitope tags occurred despite the fact that the epitopes bound by the antibodies mapped to the N terminus or N terminal portion of the protein. This finding suggests C terminal epitope tagging may interfere with function of Cthrc1, which has implications for published studies using Cthrc1 modified in this manner.

In summary, we report here that Cthrc1 is a marker for activated stromal cells and Cthrc1 expression was not detectable in the cancer cells. Furthermore, Cthrc1 is expressed in the human pituitary predominantly in the anterior lobe. Circulating levels in most healthy subjects are below 1 ng/ml but all subjects with red hair studied here had vastly higher levels. Cthrc1 plasma levels were also significantly elevated during pregnancy, in diabetes, in inflammatory and infectious conditions, acute myeloid leukemia but not solid cancers.
